# Smart Sensors for Structural Health Monitoring and Nondestructive Evaluation: 2nd Edition

**DOI:** 10.3390/s26051453

**Published:** 2026-02-26

**Authors:** Zenghua Liu

**Affiliations:** School of Information Science and Technology, Beijing University of Technology, Beijing 100124, China; liuzenghua@bjut.edu.cn

## 1. Introduction

Sensors play a vital role in nondestructive evaluation (NDE) and structural health monitoring (SHM), responsible for signal excitation and/or reception. To improve their detection capability, numerous studies have focused on configuration and performance [1,2,3,4,5,6,7,8]. This is of paramount importance, as sensor performance directly impacts the implementation and effectiveness of NDE and SHM. However, with increasingly harsh testing conditions and growing data, sensor technology faces heightened demands, including high-temperature resistance, long-term monitoring, and size constraints [9,10,11,12,13,14]. For structures with complex geometries, integrating sensors with robots to plan scanning paths and orientations has become a key research and application focus in the NDE field [15,16,17].

With the development of NDE and SHM, the widespread application of artificial intelligence (AI) to sensor arrays and big data processing has become imperative. The application of a sensor array is also essential to achieving large-scale detection or enhancing detection capacity. This inevitably involves the processing of big data and the intuitive display of detection results. The growing complexity and size of data present big challenges in data processing and computing. Meanwhile, the application of AI significantly improved data automated defect detection from ultrasonic data [18,19], presenting it as a tool through which to achieve fast and accurate analysis without human interference based on a large amount of data [20,21,22,23,24].

This Special Issue “Smart Sensors for Structural Health Monitoring and Nondestructive Evaluation: 2nd Edition” is a collection of the most recent original contributions relating to all facets of smart sensors utilized in structural health monitoring and nondestructive evaluation. The call for papers for this Special Issue sought those covering topics such as the engineering applications of NDE methods and sensing technologies, and the development of sensors with a consideration of temperature effects and applications in harsh environments.

## 2. Overview of Published Papers

In this context, this Special Issue comprises eight papers on the latest advancements in smart sensors for structural health monitoring and nondestructive evaluation. Each of these original contributions (two review papers and six research papers) underwent a rigorous peer review process, undertaken by a minimum of two expert reviewers over at least two rounds of revision. The papers published in the current Special Issue are briefly summarized as follows.

In the first contribution, the authors systematically review the current research status of pipeline in-line inspection (ILI) technologies, with a focus on four major technological systems: electromagnetic, acoustic, optical, and robotic. The operational principles, application scenarios, advantages, and limitations of each technology are analyzed in detail. It is concluded that although existing technologies have achieved significant progress in the accuracy of defect detection and environmental adaptability, they still face challenges, including insufficient adaptability to complex environments, the inherent trade-off between detection accuracy and efficiency, and high equipment costs. Future research directions are identified, including intelligent algorithm optimization for multi-physics collaborative detection, miniaturized and integrated design of inspection devices, and scenario-specific development for specialized environments. Through technological innovation and multidisciplinary integration, pipeline ILI technologies are expected to progressively realize efficient, precise, and low-cost lifecycle safety monitoring of pipelines.

The authors of the second contribution systematically review the capabilities, challenges, and practical implementations of the most widely utilized and emerging sensing technologies in SHM for infrastructure, addressing a critical research gap. While many existing reviews focus on individual methods, comprehensive cross-method comparisons have been limited due to the highly tailored nature of each technology. The authors address this by proposing a novel framework comprising five specific evaluation criteria: deployment suitability in SHM, hardware prerequisites, characteristics of the acquired signals, sensitivity metrics, and integration with Digital Twin environments. Applying this framework, both the advantages and constraints of established sensing technologies are analyzed. Critical trade-offs in scalability, environmental sensitivity, and diagnostic accuracy are also highlighted. Recognizing these challenges, the authors explore next-generation advancements, such as self-sensing structures, unmanned aerial vehicle deployment, IoT-enabled data fusion, and enhanced Digital Twin simulations. These innovations aim to overcome existing limitations by enhancing real-time monitoring, data management, and remote accessibility. This review provides actionable insights for researchers and practitioners, while identifying future research opportunities to advance scalable and adaptive SHM solutions for large-scale infrastructure.

The third contribution is an experimental study using an ultrasonic technique to investigate the impact of extremely low temperatures on the performance of lithium-ion batteries. In this experiment, lithium-ion polymer batteries were aged in three low-temperature conditions: in a temperature chamber (−34 °C), in a dry ice bath (−78 °C), and in a liquid nitrogen bath (−196 °C). While the battery aged in liquid nitrogen was damaged, the batteries aged in the chamber and dry ice bath were then subjected to charge and discharge cycles, and simultaneously monitored using the ultrasonic technique. Three key ultrasonic parameters were measured: signal amplitude, time-of-flight (TOF), and TOF shift, using 5 MHz commercial ultrasonic transducers. These measurements were conducted alongside electrical measurements to monitor the batteries throughout the testing cycles. The results showed that the aged batteries exhibited significantly lower ultrasonic amplitude compared to the control batteries. Additionally, as the cycle number increased, the TOF increased, and the discharge capacity decreased. The TOF shift increased linearly with the discharge capacity. Overall, the ultrasonic amplitude proved to be a reliable parameter for differentiating the control and low-temperature aged batteries.

In the fourth contribution, the authors investigate the potential of the unique characteristics of pulse-echo sensing systems for ultrasonic immersion testing in harsh environments. Finite element simulations and laboratory experiments are used to demonstrate the unique characteristics of pulse-echo immersion testing. Using an aluminum nitride piezoelectric element mounted on a vessel wall, characteristics associated with electrode thickness, couplant, backing material, and an acoustic matching layer are investigated. Considering a wave path through a vessel wall and into a fluid containing a target, when the travel distance in the fluid is relatively short, it can be difficult to discern the target echo from the reverberations in the vessel wall. When an acoustic matching layer between the vessel wall and the fluid does not suffice, a simple subtractive signal-processing method can minimize the reverberations, leaving just the target echoes of interest. Simulations and experiments demonstrate that sufficient target echoes are detected to determine the time-of-flight. Furthermore, a simple disc-like surface anomaly on the target is detectable.

The authors of the fifth contribution propose a composite pulse excitation technique using an air-coupled ultrasonic detection system to overcome the problems of the low signal-to-noise ratio and poor performance of wood ultrasonic images caused by ring-down vibrations during the ultrasonic quality detection of wood. Through a mathematical analysis of the output of the ultrasonic transducer, the conditions necessary for implementing composite pulse excitation were analyzed and established, and its feasibility was verified through COMSOL simulations. Firstly, wood samples with knot and pit defects were used as experimental samples. The parameters for the composite pulse excitation technique were refined by conducting A-scan measurements on both defective and non-defective areas of the samples. Moreover, two stepper motors were employed to control the path for C-scan imaging to detect wood defects. The experimental results showed that the composite pulse excitation technique significantly enhanced the precision of nondestructive ultrasonic testing for wood defects compared to the traditional single-pulse excitation method. This technique successfully achieved precise detection and location of pit defects, with a detection accuracy rate of 90% for knot defects.

In the sixth contribution, the authors develop a novel water probe for the scanning acoustic microscopy (SAM) system; that is, a water stream. During the scanning process, water was supplied using a water stream instead of immersing the sample in water, which led to a simple design of an automotive SAM system and a reduction in time consumption. In addition, using a water stream in the SAM system was shown to avoid contamination of the sample due to immersion in water for long-time scanning. The water stream was designed based on the measured focal length calculation of the transducer and simulated to investigate the internal flow characteristics. To validate the simulation results, the water stream was prototyped and applied to the TSAM-400 and W-FSAM traditional and fast SAM systems to successfully image some samples, such as carbon fiber-reinforced polymers, a printed circuit board, and a 6-inch wafer. These results demonstrate the design method of the water probe applied to the SAM system.

In an actual building setting where fifth-generation (5G) millimeter-wave communications signals are present, passive imaging of the radiation propagating through a wall defect can be conducted using interferometric processing without emitting additional signals in an already-crowded spectrum. In the seventh contribution, the authors investigate the use of millimeter-wave interferometric imaging of defects in building walls and shielded structures by capturing the transmission of 5G millimeter-wave signals through the defects. The ability to image defects was explored experimentally by capturing the transmission of 38 GHz signals through materials using a 24-element interferometric receiving array.

In the eighth contribution, to address the problems of weak geometric features, low signal response amplitude, and insufficient spatial resolvability of near-surface defects in metal substrates, a high-resolution spatiotemporal-coded eddy-current array probe is proposed. The probe adopts an array topology with time-multiplexed excitation and adjacent differential reception, achieving a balance between high common-mode rejection ratio and high-density spatial sampling. Experimental results indicate that, under a 50 kHz excitation frequency, the array eddy-current response achieves an optimal trade-off between signal amplitude and spatial geometric consistency. The proposed M-DECA probe enables high-resolution imaging and quantitative characterization of near-surface defects in metal substrates, providing an effective solution for the electromagnetic detection of near-surface, low-contrast defects.

I would like to express my deepest gratitude to the *Sensors* team for their continuous support throughout the preparation of this collection. My sincere thanks are also extended to all of the contributing authors and the anonymous expert reviewers whose invaluable efforts helped build an Issue of the utmost quality.

## 3. Conclusions

This 2nd Edition Special Issue offers a collection of eight papers addressing various topics related to the use of smart sensors for structural health monitoring and nondestructive evaluation, delineating the state of the art and the future direction of this field. We hope that the selected papers may provide useful insights into the development and application of smart sensors for SHM and NDE, providing a useful reference for future work in these rapidly evolving research and application fields.

We give our sincere thanks to the authors who contributed their research to this Special Issue, as well as the reviewers of the submitted papers who dedicated their time and expertise to providing high-quality suggestions and comments that allowed us to present a strong collection of work.
